# Importance of Intracellular pH in Determining the Uptake and Efficacy of the Weakly Basic Chemotherapeutic Drug, Doxorubicin

**DOI:** 10.1371/journal.pone.0035949

**Published:** 2012-04-26

**Authors:** Pawel Swietach, Alzbeta Hulikova, Shalini Patiar, Richard D. Vaughan-Jones, Adrian L. Harris

**Affiliations:** 1 Department of Physiology, Anatomy and Genetics, Oxford, United Kingdom; 2 Weatherall Institute of Molecular Medicine, John Radcliffe Hospital, Oxford, United Kingdom; Université Joseph Fourier, France

## Abstract

Low extracellular pH (pH_e_), that is characteristic of many tumours, tends to reduce the uptake of weakly basic drugs, such as doxorubicin, thereby conferring a degree of physiological resistance to chemotherapy. It has been assumed, from pH-partition theory, that the effect of intracellular pH (pH_i_) is symmetrically opposite, although this has not been tested experimentally. Doxorubicin uptake into colon HCT116 cells was measured using the drug's intrinsic fluorescence under conditions that alter pH_i_ and pH_e_ or pH_i_ alone. Acutely, doxorubicin influx across the cell-membrane correlates with the trans-membrane pH-gradient (facilitated at alkaline pH_e_ and acidic pH_i_). However, the protonated molecule is not completely membrane-impermeant and, therefore, overall drug uptake is less pH_e_-sensitive than expected from pH-partitioning. Once inside cells, doxorubicin associates with slowly-releasing nuclear binding sites. The occupancy of these sites increases with pH_i_, such that steady-state drug uptake can be greater with alkaline cytoplasm, in contradiction to pH-partition theory. Measurements of cell proliferation demonstrate that doxorubicin efficacy is enhanced at alkaline pH_i_ and that pH-partition theory is inadequate to account for this. The limitations in the predictive power of pH-partition theory arise because it only accounts for the pH_i_/pH_e_-sensitivity of drug entry into cells but not the drug's subsequent interactions that, independently, show pH_i_-dependence. In summary, doxorubicin uptake into cells is favoured by high pH_e_ and high pH_i_. This modified formalism should be taken into account when designing manoeuvres aimed at increasing doxorubicin efficacy.

## Introduction

The entry of weakly basic or weakly acidic molecules into cells can show pH-dependence arising from differences in the membrane permeability of their protonated and unprotonated forms [1], [2], [3]. This so-called pH-partitioning is relevant to chemotherapy because many anti-cancer drugs are protonatable and tumours typically have substantially lower extracellular pH (pH_e_) than normal tissue, whilst maintaining a modestly alkaline intracellular pH (pH_i_) [3], [4], [5], [6]. pH-partition theory has been tested experimentally by correlating drug uptake or efficacy with the trans-membrane pH gradient (ΔpH = pH_e_−pH_i_). Cellular accumulation of the weakly-basic drug doxorubicin [7] has been shown to decrease at low pH_e_, in accordance with pH-partition theory [1], [2], [8], [9]. The acidic extracellular milieu of tumours is therefore predicted to confer a degree of physiological resistance to weakly-basic drugs [9], [10], and manoeuvres that raise pH_e_ have been proposed to improve doxorubicin efficacy [1], [8]. Doxorubicin resistance may also arise from sequestration of the drug into acidic vesicles [11]. pH-partition theory has been applied to explain this phenomenon, and manoeuvres that raise intra-vesicular pH (pH_v_) have been shown to enhance doxorubicin efficacy [12], [13].

Many studies of the pH-sensitivity of doxorubicin uptake, or its cytotoxicity, have measured responses to changes in *extracellular* pH [1], [2], [8], [9]. Such manoeuvres will also change *intracellular* pH because of the pH_e_-sensitivity of transporters that regulate the cell's acid-base balance [14]. The effect of pH_i_ – independently of pH_e_ – on doxorubicin uptake has not been tested robustly. It cannot, therefore, be excluded that higher drug uptake at alkaline pH_e_ arises from higher pH_i_, in an apparent contradiction to pH-partition theory. Recent *in vitro* work has shown that doxorubicin forms an adduct with DNA [15] in a pH-sensitive manner [16], decreasing as pH is reduced below 7. This process may introduce physiologically-relevant pH_i_-sensitivity of doxorubicin uptake into cells, independently of pH-partitioning across the membrane. Attempts have been made to disentangle the effects of pH_i_ and pH_e_ on drug uptake by comparing cells with different pH_e_−pH_i_ relationships, for example, by comparing low-pH adapted CHO cells with control cells [2]. Comparison between phenotypically-distinct sub-populations can introduce additional variables that may affect drug uptake. For instance, at pH_e_ = 7.4, when both normal and low-pH adapted cells have similar pH_i_, doxorubicin uptake was much higher in the former. Correlations between ΔpH and drug uptake, deemed as evidence for the pH-partition theory, have typically been made over a range of negative ΔpH (i.e. pH_e_<pH_i_), but not extended to symmetrically positive ΔpH. In these studies, changes to pH_i_ have typically been much smaller than the associated changes to pH_e_, making it difficult to characterise the importance of pH_i_ in drug uptake. Similarly, doxorubicin uptake into vesicles has been investigated in the context of changing pH_v_ at near-constant pH_i_ [12], [13]. Further testing of pH-partition theory would require studies performed under conditions where pH_i_ is manipulated to a greater degree than pH_e_ or pH_v_.

In this study, we use doxorubicin's intrinsic fluorescence to study the drug's uptake and accumulation over a range of pH_i_ and pH_e_ values, manipulated by changing bathing medium pH (to alter pH_i_ and pH_e_ in parallel), applying weak-acids at constant pH_e_ (to alter pH_i_) or inhibiting membrane-bound acid-base transporters (to alter pH_i_ at constant pH_e_). We then test the inferences made from isolated cells on multi-cellular 3-D spheroids [17], [18]. Finally, the effects of changing pH_i_ with pH_e_ or pH_i_ alone on doxorubicin efficacy were tested using an index of proliferation. P53 wild-type human colon HCT116 cells were used in the study, as these have relatively low expression levels of the drug resistance-conferring P-glycoprotein [19], [20] and the proposed doxorubicin carrier SLC22A16 [21]. In addition, these cells have well-characterised pH regulation, little acid-extrusion by H^+^ ATPase pumps (i.e. Na^+^- and HCO_3_^−^-independent flux) and can form multi-cellular spheroids [22]. Experimental data were analysed with a mathematical model to dissect the relationships between pH_i_, pH_e_ and drug uptake.

Our results indicate that pH-partition theory alone is inadequate to fully account for the pH-sensitivity of doxorubicin uptake. Firstly, we find that the protonated form of doxorubicin can cross membranes, albeit slower than the unprotonated form. Secondly, once inside the cell, the drug associates with nuclear binding sites in a pH-dependent manner. We conclude that the behaviour of free doxorubicin can be approximated by the pH-partition theory, but its intracellular accumulation requires the introduction of a pH_i_-sensitive drug-binding process. We propose that doxorubicin accumulation is favoured by a combination of high pH_e_ *and* high pH_i_.

## Methods

### Solutions

All solutions were based on Hepes/Mes-buffered normal Tyrode (125 mM NaCl, 4.5 mM KCl, 20 mM Hepes, 20 mM Mes, 1 mM CaCl_2_, 1 mM MgCl_2_, pH adjusted to 7.4). To change pH_e_ (and, secondarily, pH_i_), solution pH was titrated to 6.4 or 6.8 or 7.8 (whilst maintaining osmolarity by adjusting NaCl). To change pH_i_ at constant pH_e_: *(i)* NaCl was iso-osmotically replaced with 40 mM or 80 mM NaAcetate (reduces pH_i_), *(ii)* Na^+^ was replaced with N-methyl-D-glucamine (inactivates NHE and allows CHE to acid-load cell), *(iii)* 5-(N,N-dimethyl) amiloride (DMA) was added (inhibits NHE and allows CHE to acid-load cell), *(iv)* Cl^−^ was replaced with gluconate (inactivates CHE and allows NHE to base-load cells), or *(v)* Hepes/Mes was replaced with CO_2_/HCO_3_^−^ (alters the steady-state pH_e_−pH_i_ relationship by activating additional pH_i_ regulating transporters).

### Imaging isolated cells under superfusion

Colon cancer HCT116 cells (provided by Cancer Research UK; [17], [23]) were grown in Dulbecco's Modified Eagle's Medium (DMEM) containing NaHCO_3_, in an atmosphere of 5% CO_2_ for 48–72 hours until confluency. Prior to experiments, cells were re-suspended in Hepes-buffered DMEM (for no more than 3 hours). A 200 µl-aliquot of cell-suspension was applied to a poly-L-lysine pre-treated coverslip, mounted at the base of a Perspex superfusion chamber. After 10 minutes to allow for cell-adhesion (and fluorescent dye-loading, where applicable), cells were superfused with solution heated to 37°C at 2 ml/min [22]. By switching between two solution lines, it was possible to change the superfusate with a time-constant of <8 sec. pH_i_ was measured in cells loaded with carboxy-SNARF-1 (excitation 514 nm, fluorescence emission collected at 580 nm and 640 nm). The emission ratio was calibrated in units of pH by the ‘nigericin’ technique [24]. In separate experiments, doxorubicin (50 µM) uptake was measured using its intrinsic fluorescence (excitation 488 nm, emission 580 nm). This signal was not significantly pH-sensitive (Fig S1A), i.e. the protonated and unprotonated forms are equally fluorescent. By confocally imaging an optical slice, it is possible to measure fluorescence within cells and in the extracellular solution simultaneously.

### Measurements with flow cytometry

Cells were pre-equilibrated for 1 hour with a desired Hepes/Mes-buffered solution to attain steady-state pH_i_. Cells were then treated with doxorubicin (50 µM) for a further 2 hours. Drug accumulation in cells was quantified with a Beckman Coulter Quanta SC flow cytometer (excitation 488 nm; emission 580 nm) in drug-free medium. Measurements were limited to 5 minutes and back-extrapolated to account for slow release of doxorubicin in drug-free bathing media. To measure pH_i_, cells were subjected to the same procedure (with the exception of doxorubicin treatment) and AM-loaded with carboxy-SNARF-1 for 10 minutes before flow-cytometry (excitation 488 nm; emission 580 nm and 640 nm).

### Culturing and imaging spheroids

HCT116 cells were cultured in McCoy's 5A medium. Aggregation into spheroids was initiated by plating 4×10^6^ cells in 250 mL spinner flasks (Techne MCS, UK) spun at 40 rpm for 2–9 days. Spheroids were superfused with buffer containing doxorubicin (50 µM) for 2 hours. To correct for the depth-dependent decrease in light-emission, doxorubicin fluorescence was imaged alternately with the pH-insensitive, UV-excitable and spectrally-resolvable dye, 7-amino-4-methyl coumarin (AMC; 30 µM). Extracellular pH within the spheroid mass was measured with fluorescein sulphonic acid (30 µM) [17].

### Nuclear vs cytoplasmic doxorubicin fluorescence

HCT116 cells were grown as confluent monolayers and treated with doxorubicin (50 µM) and the nuclear stain Hoechst 33342 (10 µg/ml) for 2 hours. Fluorescence from doxorubicin (>580 nm) and from Hoechst (<480 nm) were excited at 488 nm and 405 nm, respectively. Doxorubicin fluorescence was summed in nuclear regions (identified by Hoechst fluorescence) and non-nuclear regions (with sub-threshold Hoechst fluorescence). See Figure S2 for details of the image-processing method.

### Proliferation assay for determining doxorubicin efficacy

1.0×10^5^ cells were seeded per well in 96-well plates and incubated for 48 hours. Confluent cells at the base of the wells were washed with PBS twice and then bathed in Hepes/Mes-buffered normal Tyrode solution at pH = 7.4 (the control) or one of five solutions that alter pH_i_: *(i–ii)* normal Tyrode titrated to pH 6.4 or 7.8 to alter pH_e_ and pH_i_, *(iii–v)* normal Tyrode at pH 7.4 containing DMA or lacking Na^+^ salts or Cl^−^ salts to alter pH_i_ at constant pH_e_ (NB: these manoeuvres change the balance of trans-membrane acid/base fluxes, which alters pH_i_ but not pH_e_ because the bulk extracellular volume is much larger than intracellular volume). After allowing 45 minutes for cells to re-equilibrate their trans-membrane pH gradient, solutions were replaced with ones containing doxorubicin (1 to 300 µM). After 2 hours of drug treatment, cell proliferation was assessed using the CellTiter Blue assay (Promega, UK). Proliferation was calculated as the ratio of absorbance at 562 nm to 595 nm, normalized to that ratio in the absence of doxorubicin (this will take into account doxorubicin-independent differences in cell-survival in the six bathing media used). At >550 nm, absorbance due to doxorubicin is negligible.

### Mathematical model of doxorubicin uptake

The model assumes that doxorubicin fluorescence is *(i)* proportional to its concentration and *(ii)* a linear combination of free and bound doxorubicin. The drug's pK was taken as 8.2 [1], [2], [8], [9]. Best-fitting was used to estimate the permeability constant of the protonated and unprotonated forms (P_HDox_, P_Dox_), the maximal binding capacity of the ‘receptor’ (B_max_) and its association and dissociation constants (k_on_, k_off_). It was assumed that both forms of drug bind equally to the ‘receptor’. The pH-sensitivity of drug binding was modelled by scaling B_max_ (i.e. non-competitive; acid-dissociation constant K_H_) or by scaling k_on_ (i.e. competitive; acid-dissociation constant Q_H_). The equations describing the concentration of free doxorubicin ([DOX]) and bound doxorubicin ([DOX*]) inside cells are:
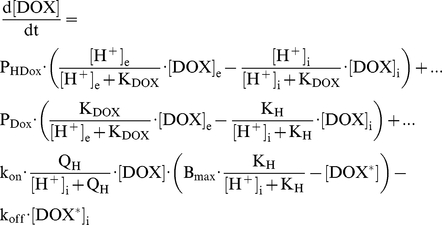


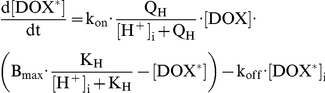



## Results

### Intracellular doxorubicin associates with a slowly-releasing binding site

**Figure 1:**
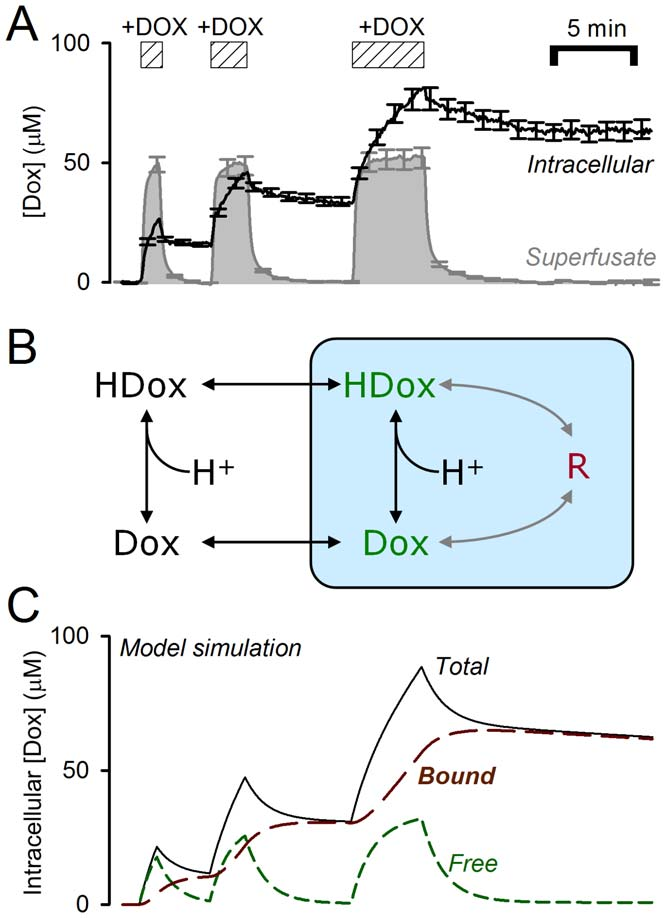
Intracellular doxorubicin associates with a slowly-releasing intracellular binding site. (A) HCT116 cells superfused with Hepes/Mes buffer at pH = 7.4, 37°C. Doxorubicin (DOX; 50 µM) was applied transiently by switching rapidly between drug-free and drug-containing solution (average of 25 cells, ±SEM). (B) Proposed model with equilibria involving free and bound doxorubicin. (C) Mathematical simulation showing the fast rise of intracellular doxorubicin upon exposure, and its slow release upon reversal of the trans-membrane concentration gradient.

Colon HCT116 cells (resting pH_i_ = 7.2) were exposed transiently to solution (pH_e_ = 7.4) containing 50 µM doxorubicin for 1, 2 and 5 minutes (Fig. 1A). This dose is adequately fluorescent, potently cytotoxic over a broad pH_e_ range [9] and can produce significant binding to DNA for a more accurate characterisation of drug accumulation in cells [16], [25]. Doxorubicin wash-out was considerably slower than its uptake. This asymmetry cannot be explained in terms of the trans-membrane pH gradient (ΔpH of only +0.2). The rise of cell-averaged fluorescence above the extracellular background suggests that the drug concentrates inside cells [11], [13], [15], [26]. The experimental time-courses can be simulated by accounting for doxorubicin-binding to an intracellular ‘receptor’ (Fig. 1B) of fast association (k_on_) and slow dissociation (k_off_) kinetics (Fig. 1C). Best-fitting values for the receptor's maximal binding capacity (B_max_), ‘on’ and ‘off’ rate constants (k_on_ and k_off_) were 506 µM, 32.5 s^−1^×M^−1^ and 2.345×10^−4^ s^−1^, respectively, giving a doxorubicin binding constant of 7.2 µM. Total intracellular doxorubicin concentration is therefore given by the sum of ‘free’ and ‘bound’ drug.

### Low extracellular pH decreases the rate of doxorubicin uptake and accumulation in isolated cells

**Figure 2:**
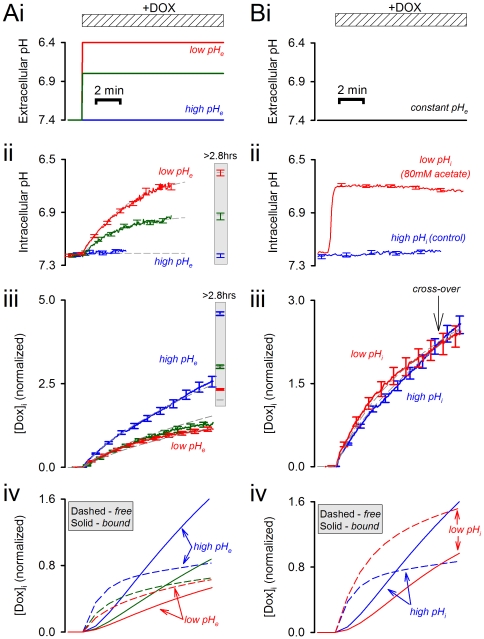
Effect of changing extracellular and intracellular pH on doxorubicin uptake. Time-courses show the average (±SEM) of at least 25 cells. (A) *(i)* Extracellular pH was changed by switching to superfusates titrated to pH 6.8 or 6.4, simultaneously with the application of 50 µM doxorubicin. *(ii)* Intracellular pH measured in separate experiments using carboxy-SNARF-1. *(iii)* Intracellular doxorubicin, normalized to its extracellular signal. Inset shows intracellular fluorescence at steady state, attained after 2.8 hours of drug-exposure. *(iv)* Simulated doxorubicin time-courses. (B) *(i)* Intracellular pH was reduced to 6.7 at constant extracellular pH by superfusing cells with 80 mM acetate in the presence of the Na^+^/H^+^ exchange inhibitor, dimethyl amiloride (DMA; 30 µM). Doxorubicin was applied once pH_i_ attained a steady-state. *(ii)* Intracellular pH measured with carboxy-SNARF-1. *(iii)* Intracellular doxorubicin fluorescence, showing the cross-over of time-courses for pH_i_ = 7.2 and 6.7. *(iv)* Simulation of doxorubicin-time-courses.

HCT116 cells were exposed to doxorubicin at different superfusate pH (Fig. 2A) to measure the drug's initial uptake rate and its steady-state accumulation as a function of the trans-membrane pH gradient. Figure 2Ai illustrates the experimental protocol and Figure 2Aii shows the pH_i_ response to changes in pH_e_. For measurements of the initial rate of drug uptake, it was assumed that starting pH_i_ is 7.2. Analyses of doxorubicin accumulation at steady-state must, however, take into account the slow, pH_e_-evoked changes in pH_i_.

**Figure 3:**
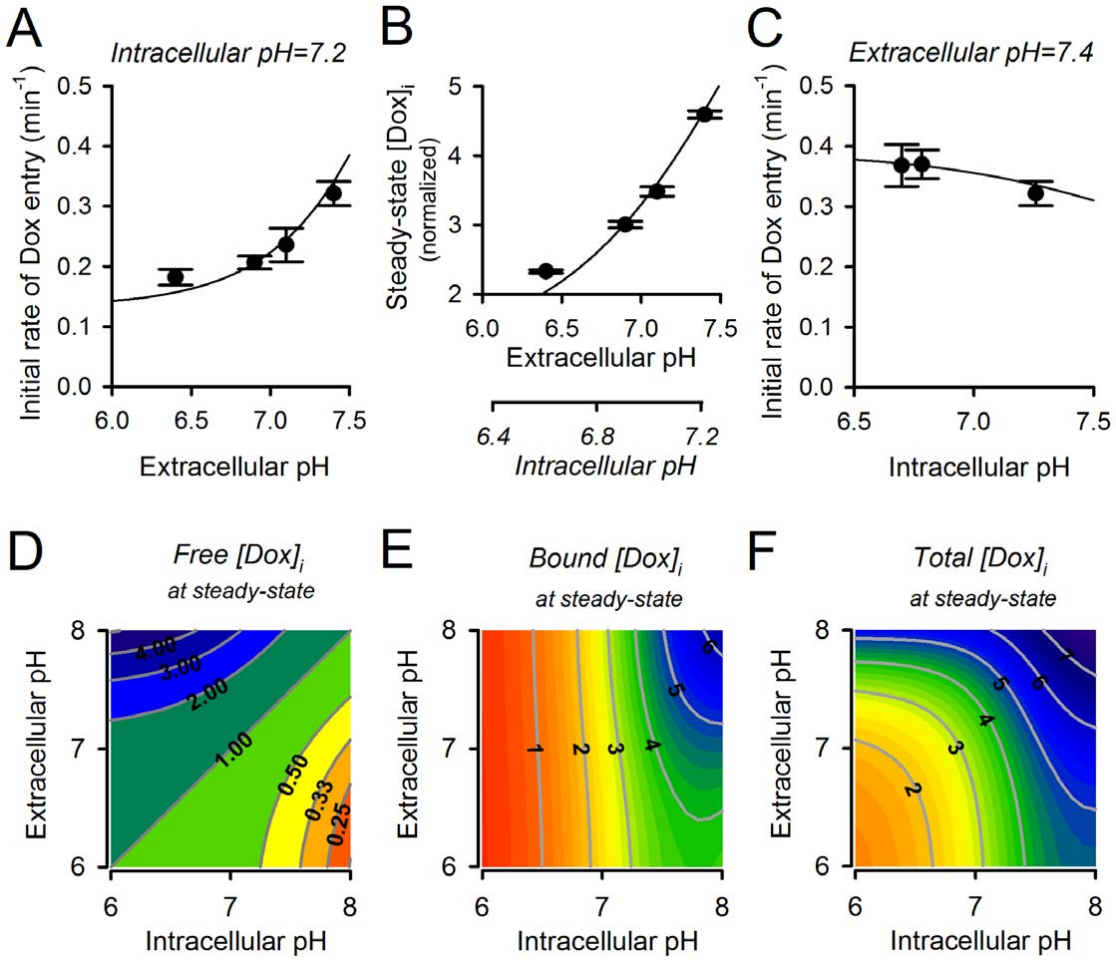
Effect of intracellular and extracellular pH on drug uptake and accumulation. (A) Initial rate of doroxubicin uptake, measured at constant intracellular pH, over a range of extracellular pH values (data from Fig. 2A) with best-fit. (B) Intracellular doxorubicin at steady-state, normalized to extracellular concentration, over a range of extracellular pH values. Secondary axis plots steady-state pH_i_ attained at given pH_e_. (C) Initial rate of doxorubicin uptake, measured at constant extracellular pH, over a range of intracellular pH values (data from Fig. 2B) with best-fit. Model predictions for the steady-state relationship between intracellular pH, extracellular pH and either (D) free, (E) bound or (F) total doxorubicin. Contour labels denote total intracellular doxorubicin concentration, normalized to its extracellular concentration (50 µM).

In agreement with pH-partition theory, doxorubicin uptake was slowest at acidic pH_e_ (Fig. 2Aiii). The mathematical model was used to best-fit these experimental data for the permeability constants of the protonated and unprotonated forms of drug (P_HDox_; P_Dox_). If only the unprotonated form of drug were able to enter cells, drug uptake at pH_e_ = 6.4 should be ∼9-fold lower than at pH_e_ = 7.4. Our data suggest that doxorubicin uptake is less pH_e_-sensitive than this: uptake at pH_e_ = 6.4 (ΔpH = −0.8) was only 1.7-fold lower than at pH_e_ = 7.4 (ΔpH = +0.2) (Fig. 3A). This would be expected if the protonated drug could also permeate across cell-membranes. The pH_e_-sensitivity of drug uptake can be simulated with a P_Dox_ of 0.041 s^−1^ and a 12-fold lower P_HDox_. In summary, doxorubicin protonation does not render it completely membrane-impermeant. Consequently, extracellular acidification reduces the initial rate of drug uptake to a smaller degree than predicted by pH-partition theory.

Intracellular doxorubicin accumulation was measured after 2.8, 3.3 and 7.5 hours and was found to attain a steady-state by 2.8 hours. Steady-state drug accumulation was lowest at acidic pH_e_ (Fig. 2Aiii). During the course of this experiment, the trans-membrane pH gradient gradually decreases because pH_i_ tends towards pH_e_ (Fig. 2Aii). According to pH-partition theory, the time-courses of doxorubicin accumulation measured at different pH_e_ should gradually converge, having attained the greatest separation initially, before the onset of the secondary pH_i_ change. The experimental data are contrary to this (see Fig. 3A vs 3B): the initial rate of drug uptake was 1.7-fold lower at pH_e_ = 6.4 (ΔpH = −0.8) than at pH_e_ = 7.4 (ΔpH = +0.2), but at steady-state, drug accumulation was 2.0-fold lower at the more acidic pH_e_ (despite the narrowing of ΔpH to −0.2). The on-going intracellular acidification at low pH_e_ *reduces* doxorubicin accumulation in cells (Fig. 2Aiii). While this is incompatible with pH-partition theory, it could be explained by reduced doxorubicin binding to ‘receptors’ at acidic pH_i_. These data fit best to a model with pH_i_-dependence of binding capacity B_max_ (i.e. non-competitive model) rather than of affinity (i.e. competitive model). The acid-dissociation constant (K_H_) that best describes the data was estimated to be 10^−7.44^ (Fig. 2Aiv).

### Low intracellular pH increases the rate of doxorubicin uptake in isolated cells

pH-partition theory was tested further by comparing drug-uptake at reduced pH_i_ but unchanged pH_e_ (Fig. 2Bi–ii). This was attained by applying superfusates (pH = 7.4) containing 80 mM sodium acetate (to acid-load the cytoplasm) and 30 µM DMA (to block acid-extrusion that would otherwise ensue). Acidification, when confined to the intracellular compartment, increased the initial rate of drug uptake, as expected from pH-partition theory (Fig. 3C). However, after 11 min of exposure, drug accumulation became larger at higher pH_i_ values (Fig. 2Biii). An analogous cross-over was observed with 40 mM sodium acetate at 14 minutes. This behaviour is not predicted by the pH-partition theory, but could be explained by reduced drug-binding to the intracellular ‘receptor’ at low pH_i_, as proposed herein. Model simulations were again in good agreement with the data (Fig. 2Biii).

### Intracellular pH is a principal determinant of doxorubicin accumulation in its slowly-releasable form

The parameterised mathematical model was used to simulate the steady-state concentration of ‘free’ and ‘bound’ doxorubicin as a function of pH_i_ and pH_e_ (Fig. 3D, E, F) The trans-membrane distribution of *free* doxorubicin is in agreement with pH-partition theory, being greatest at low pH_i_ and high pH_e_ (Fig. 3E). The concentration of *bound* doxorubicin increases with pH_e_ (due to increased influx of drug) and with pH_i_ (due to increased binding of drug). Overall, optimal doxorubicin uptake is attainable at high pH_i_ *and* pH_e_. This was tested further using flow cytometry [27].

**Figure 4:**
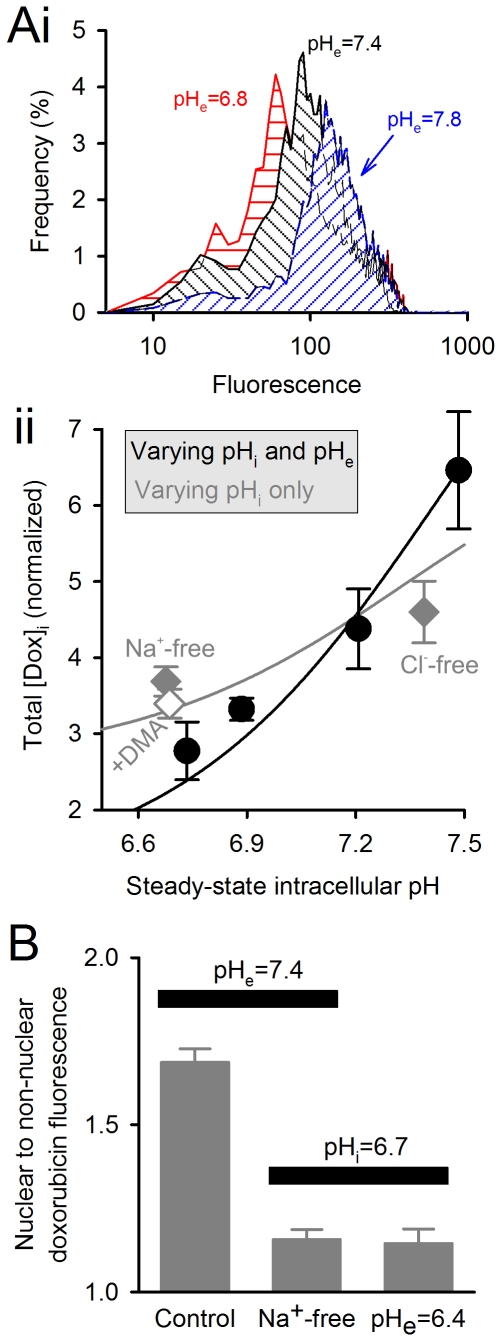
Importance of intracellular pH in determining doxorobucin accumulation. (A) *(i)* Specimen histogram of intracellular doxorubicin fluorescence (from >5000 cells) at different extracellular pH. *(ii)* Plot of intracellular doxorubicin (±coefficient of variation) versus intracellular pH. *Black circles:* intracellular pH manipulated by varying extracellular pH. *Grey symbols:* intracellular pH manipulated at constant extracellular pH. (B) Data from HCT116 monolayers treated with doxorobicin and Hoechst 33342. Ratio of doxorubicin fluorescence in nuclear (Hoechst 33342 positive) and non-nuclear regions quantifies the degree of drug accumulation in the nucleus.

The pH_i_ of cells was manipulated by changing pH_e_ or by altering the balance between acid-loading and acid-extruding membrane transporters, at constant pH_e_. In Hepes/Mes-buffered (i.e. CO_2_/HCO_3_^−^-free) media, resting pH_i_ in HCT116 cells is set by the balance of acid-extruding Na^+^/H^+^ exchange (NHE) and acid-loading Cl^−^/OH^−^ exchange (CHE) [22]. By replacing extracellular Na^+^ isosmotically with N-methyl-D-glucamine or by including the NHE inhibitor DMA (50 µM), CHE-flux drives pH_i_ to a more acidic level. Conversely, pH_i_ can be driven in the alkaline direction by NHE, when CHE is blocked by replacing extracellular Cl^−^ isosmotically with gluconate. After allowing 1 hour for the attainment of a new steady-state pH_i_, HCT116 cells were treated with 50 µM doxorubicin for 2 hours. Figure 4Ai shows doxorubicin fluorescence histograms for three individual flow cytometric experiments. Parallel experiments were performed in the absence of doxorubicin, on cells loaded with carboxy-SNARF-1 to measure steady-state pH_i_. Figure 4Aii plots the measured and simulated relationship between pH_i_ and total intracellular doxorubicin. In agreement with the mathematical predictions, but in contradiction to pH-partition theory, doxorubicin accumulation increases with pH_i_, and is further augmented by raised pH_e_.

Doxorubicin, like other anthracyclines, targets DNA [15], [25], [28], [29]. The possibility that the nucleus hosts the pH_i_-sensitive, slowly-releasing ‘receptor’ was tested by measuring doxorubicin fluorescence in nuclear and extra-nuclear regions. Nuclear regions were identified by using the DNA-binding stain, Hoechst 33342 (Figure S2). HCT116 monolayers were equilibrated with solutions at pH_e_ = 7.4 (the control), pH_e_ = 6.4 (to reduce pH_i_ and pH_e_) and Na^+^-free solution at pH_e_ = 7.4 (to reduce pH_i_ at constant pH_e_). Monolayers were then treated with doxorubicin and Hoechst 33342 for two hours and then imaged confocally. Doxorubicin was distributed near-uniformly in non-nuclear regions, suggesting that the degree of sequestration into acidic compartments is small in HCT116 cells. Nuclear sequestration of doxorubicin, expressed as a ratio of total doxorubicin fluorescence in nuclear to non-nuclear regions, was markedly reduced at low pH_i_, irrespective of whether this was attained by reducing pH_e_ or removing extracellular Na^+^ (Fig. 4B). In summary, the distribution of doxorubicin between cytoplasm and the nucleus is pH_i_-sensitive.

### Imaging uptake of doxorubicin in multi-cellular spheroids

**Figure 5:**
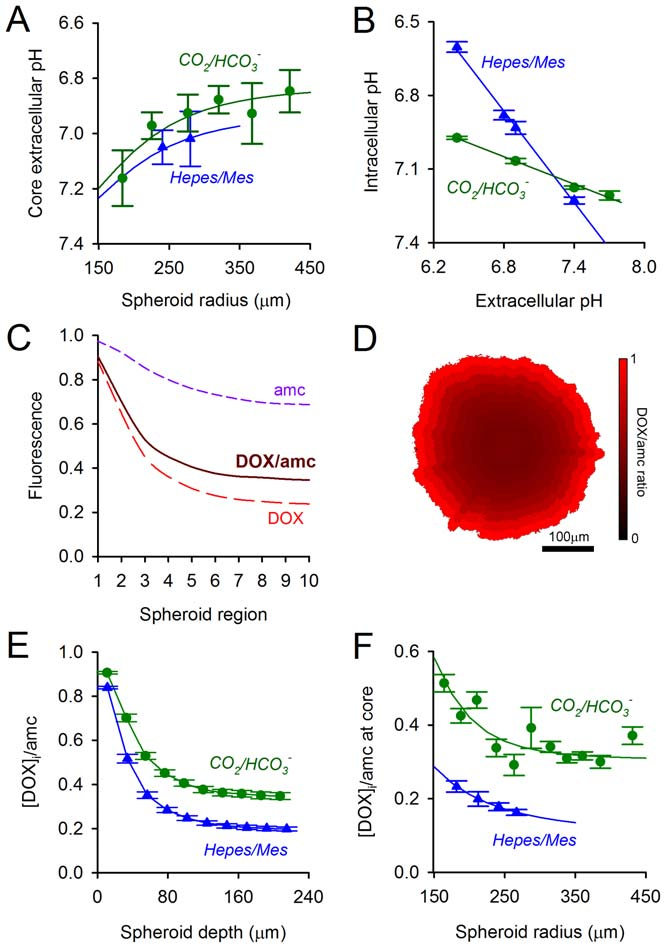
Doxorubicin accumulation in multi-cellular spheroids. (A) Extracellular pH measured at the core of spheroids, as a function of radius, under two buffering regimes: 20 mM Hepes/Mes and 5% CO_2_/22 mM HCO_3_^−^. (B) Relationship between extracellular and intracellular pH in single cells, under the two buffering regimes. (C) Radial gradients of doxorubicin and 7-amino-4-methyl coumarin: the ratio of these signals provides a measure of doxorubicin uptake corrected for light-path and absorbance. (D) Ratiometric image showing doxorubicin accumulation in a HCT116 spheroid, averaged in ten concentric layers. (E) Doxorubicin accumulation decreases with spheroid depth, as expected from the depth-dependent decrease in extracellular pH. Doxorubicin uptake is lower in Hepes/Mes-treated spheroids, i.e. at lower intracellular pH. (F) Doxorubicin uptake at the core of spheroids decreases with spheroid-size and is further reduced by replacing CO_2_^/^HCO_3_^−^ buffer with Hepes/Mes.

HCT116 cells were cultured as multi-cellular 3-D spheroids of radius 170 to 450 µm. The extracellular spaces within the spheroid-mass restrict the diffusion of metabolically-produced acids. This establishes radial gradients of pH_i_ and pH_e_, with the lowest levels attained at the spheroid-core. Figure 5A shows the relationship between spheroid radius and its core-pH_e_ (measured with fluorescein sulphonic acid). The extracellular space was buffered by CO_2_/HCO_3_^−^ or a mixture of Hepes+Mes, supplied by the superfusate as 5% CO_2_/22 mM HCO_3_^−^ or 20 mM Hepes+20 mM Mes, respectively. These solutions provide a similar level of mobile buffering capacity, resulting in comparable pH_e_ gradients (Fig. 5A). However, the presence of CO_2_/HCO_3_^−^ buffer activates additional acid-base transporters (Na^+^-HCO_3_^−^ co-transport and Cl^−^/HCO_3_^−^ exchange) which provide tighter pH_i_ control. This is illustrated by the relationship between pH_e_ and pH_i_ measured in single cells under the two buffering regimes (Fig. 5B). Replacement of Hepes/Mes buffer with CO_2_/HCO_3_^−^ will raise pH_i_ at the pH_e_ levels recorded in spheroids [17], [18]. pH-partition theory predicts that this should *decrease* doxorubicin uptake.

To measure doxorubicin uptake, spheroids were treated with drug for 2 hours and then imaged for doxorubicin fluorescence. Imaging alternately with 7-amino-4-methyl coumarin (AMC) corrected for the depth-dependent loss of signal (Fig. 5C, D). Drug uptake decreased with spheroid-depth, as expected from the radially-decreasing pH_e_ (Fig. 5E, F). In contradiction to pH-partition theory, replacement of Hepes/Mes with CO_2_/HCO_3_^−^ *increased* drug uptake for size-matched spheroids (Fig. 5E, F). This observation is, however, consistent with the notion that alkaline pH_i_ facilitates doxorubicin accumulation onto nuclear binding sites.

### Effect of intracellular and extracellular pH on doxorubicin efficacy

Doxorubicin efficacy was quantified in terms of the dose required to reduce cell proliferation by 50% (EC_50_). The effect of intra- and extracellular pH on EC_50_ was determined in cells equilibrated in solutions that alter pH_i_ with pH_e_, or pH_i_ alone. For control experiments, cells were incubated in normal Tyrode at pH 7.4. To alter pH_e_ and pH_i_, cells were incubated in normal Tyrode titrated to pH 6.4 or 7.8. To alter pH_i_ alone (pH_e_ constant at 7.4), cells were incubated in normal Tyrode containing 50 µM DMA or in solutions lacking Na^+^ or Cl^−^ salts.

**Figure 6:**
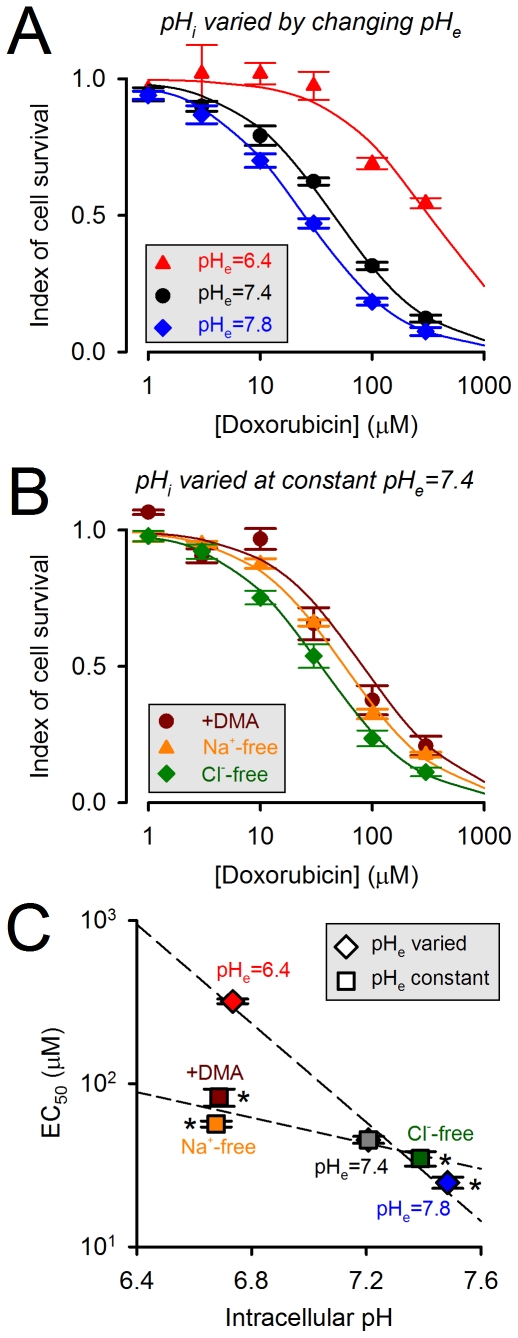
Doxorubicin efficacy as a function of intracellular pH. Cell proliferation was measured using the CellTiter blue assay kit (quantified as a ratio of absorbance at 562 nm and 595 nm). Doxorubicin efficacy was determined from dose-response curves as the concentration which results in a 50% decrease in proliferation (EC_50_). EC_50_ was measured under six different conditions that change pH_i_, with or without an associated change in pH_e_. (A) Determining EC_50_ under incubation with normal Tyrode solutions titrated to pH 7.4 (the control), 6.4 or 7.8. Incubation under these conditions also changes pH_i_ (n = 8 each). (B) Determining EC_50_ under incubation with solutions at pH_e_ = 7.4 containing 50 µM DMA or lacking Na^+^ salts or Cl^−^ salts (n = 8 each). These manoeuvres change pH_i_ by altering the balance of acid/base fluxes across membranes (but do not alter pH_e_ significantly because of the dilution effect into the large extracellular volume). (C) EC_50_ plotted against pH_i_ (see Fig. 4Aii). Alkaline pH_i_ increases doxorubicin efficacy (decreases EC_50_).

Figure 6A show the effect of changing pH_i_ and pH_e_ on the doxorubicin dose-response curve. The efficacy of doxorubicin as a cytotoxic drug increases (i.e. EC_50_ falls) as pH_e_ and pH_i_ are raised in tandem. Under pH-partition theory, changes in pH_i_ and pH_e_ should exert opposite effects on EC_50_. However, changes in pH_e_ evoke changes in pH_i_ that are parallel but of smaller magnitude (Fig. 5B). Consequently, the effects on EC_50_, shown in Figure 6A, are likely to be dominated by the pH_e_-effect, and are consistent with pH-partition theory (i.e. increasing pH_e_ increases doxorubicin membrane-permeability).

According to pH-partition theory, an increase in pH_i_ (at constant pH_e_) should *decrease* doxorubicin efficacy (i.e. raise EC_50_). However, the experimental data shown in Figure 6B are contrary to this and, instead, are supportive of the model proposed herein featuring pH_i_-dependent binding of doxorubicin to its nuclear target. Figure 6C plots EC_50_ values as a function of pH_i_ attained with the six experimental protocols, and shows that doxorubicin efficacy increases (i.e. EC_50_ falls) as pH_i_ is raised. This relationship was steeper in experiments where pH_i_ and pH_e_ were changed in parallel. This is because changes in pH_i_ and pH_e_ have synergistic effects on doxorubicin uptake and its efficacy. In summary, doxorubicin's efficacy as a cytotoxic drug depends on pH_i_ and pH_e_ in a manner predicted by the present model. These data also illustrate the inadequacy of pH-partition theory in predicting the effects of pH_i_ on drug efficacy.

## Discussion

### Evaluation of pH-partition theory

pH-partitioning has been the principal theoretical framework for describing the uptake of weakly acidic or basic drugs into cells. Its predictions have been applied, with success, to explain doxorubicin resistance in cancer cells. Doxorubicin efficacy has been shown to increase with a rise in pH_e_, although some investigations have suggested that the increase is less than predicted from pH-partitioning alone [10]. Doxorubicin flux on SLC22A16 [30] and MDR proteins [31] may produce discrepancies from pH-partitioning, particularly if these fluxes are pH-sensitive. However, the present work tested pH-partition theory on colon HCT116 cells, a cell line with only modest SLC22A16 and MDR1 expression [19], [20], [21].

Our experimental data are in partial agreement with pH-partition theory. The initial rate of doxorubicin uptake (before significant intracellular binding can occur) is favoured by a positive trans-membrane pH gradient (pH_e_−pH_i_>0; Fig. 3D). However, the measured uptake rates are less pH_e_-sensitive than expected (Fig. 3A, C). We have inferred this to be evidence that the protonated form of drug is not absolutely membrane-impermeant, but instead can enter cells, albeit 12-fold slower than the unprotonated drug. More substantial discrepancies between pH-partitioning predictions and our data arise from drug-binding to slowly-reversible (Fig. 1), pH_i_-sensitive (Fig. 2A) ‘receptors’ that are most likely nuclear (Fig. 4B). Our estimated affinity constant for doxorubicin (7.2 µM) is in the range determined previously for DNA [28], [29] and the estimated maximal binding capacity is equivalent to 3×10^8^ base-pairs (every tenth base-pair of human DNA). Our data cannot determine unequivocally if these binding sites are selective for either protonated or unprotonated drug, but the considerable difference between estimated receptor pK_H_ (7.44) and doxorubicin pK (8.2) argues against selectivity. Based on the goodness-of-fit to model simulations, our experimental data support a non-competitive model of pH-sensitivity (i.e. H^+^ ions reducing receptor availability for drug-binding). Since nuclear pH is in communication with cytoplasmic pH, drug-binding will respond to changes in pH_i_ rather than ΔpH. Importantly, the analysis presented in Figure 3F suggests that drug accumulation is similarly sensitive to changes in either pH_i_ or pH_e_ from ‘physiological’ levels of 7.2 and 7.4. The importance of pH_i_ in determining doxorubicin efficacy is illustrated experimentally in the cell proliferation assay (Fig. 6). The evidence for increased drug uptake and efficacy at alkaline pH_i_ is contradictory to pH-partitioning and emphasises the necessity of implementing our novel model for describing the effect of pH on doxorubicin resistance.

### Limitations of using fluorescence as a measure of concentration

The use of fluorescence intensity as a measure of doxorubicin concentration may introduce errors due to dye self-quenching or pH-sensitivity of excitation or emission spectra. These potential artefacts are unlikely to affect the interpretation of our data. Firstly, confocal imaging of cells at the base of a superfusion chamber minimises self-quenching in the optical slice. Nonetheless, if doxorubicin fluorescence were to saturate at high drug-concentrations, it would exacerbate the discrepancy between our data (Fig. 2) and pH-partition theory, and support the herein proposed model. Secondly, doxorubicin fluorescence is only weakly pH-sensitive (Figure S1A). Fluorescence intensity may change independently of concentration when doxorubicin dissolves into a medium of different dielectric constant, such as a non-aqueous environment. The doxorubicin emission spectrum carries a signature, quantified in terms of the 580 nm/640 nm fluorescence ratio, that correlates with the properties of the solvent [32]. Our measurements of this ratio under different bathing conditions show no significant pH-related change in the drug's environment (Figure S1B). Collectively, these findings suggest that fluorescence is a reasonable measure of doxorubicin concentration.

### Conclusions

The efficacy of doxorubicin (and potentially other related drugs) is pH-dependent because of *(i)* pH-partitioning across cell-surface and vesicular membranes (which determines drug entry and its organellar distribution) and *(ii)* the pH-sensitivity of binding to nuclear sites (which sequesters doxorubicin in a slowly-releasable form). Our study shows that changes to pH_i_ exert opposite effects on trans-membrane drug entry and on drug accumulation in nuclear sites, but the latter emerges as dominant (per pH-unit) and can determine overall efficacy, at least in HCT116 cells. These influences should be considered carefully when addressing resistance to chemotherapy. There have been three proposed pH-interventions for increasing doxorubicin efficacy. The first is alkalinisation of the extracellular space (e.g. titration to a higher pH_e_ or infusion of HCO_3_^−^) which raises pH_e_ and pH_i_ [1], [8]. The second manoeuvre is to inhibit surface membrane-bound proteins that acidify the extracellular space, such as extracellular-facing carbonic anhydrases, Na^+^/H^+^ exchangers or Na^+^-HCO_3_^−^ co-transporters [33], [34] and thereby increase pH_e_. The third intervention proposes to inhibit vesicular acidification, which reduces drug sequestration into endosomes [12], [13]. pH-partition theory predicts that the second and third manoeuvres should produce the more powerful effect on overcoming pH-related chemo-resistance, because these raise pH_e_ or pH_v_ and decrease pH_i_ (i.e. tending to reverse the trans-membrane pH-gradient). However, the present findings argue that the first manoeuvre will have a more potent effect on improving doxorubicin uptake because of the parallel rise in pH_i_ and pH_e_. It is noteworthy that inhibitors of acid-extruding proteins, proposed as anti-cancer drugs [33], may decrease doxorubicin efficacy by reducing pH_i_, if the associated change in pH_e_ is relatively small.

## Supporting Information

Figure S1 **Characterising doxorubicin fluorescence.** *(A)* Doxorubicin (50 µM) fluorescence was measured in Hepes/Mes buffered solution over a range of pH. The pH-sensitivity of fluorescence emission is only mildly pH-sensitivity (<5% per pH unit). *(B)* Intracellular doxorubicin fluorescence ratio measured flow cytometrically at 580 nm and 640 nm. This ratio carries a signature that describes changes to the ambient environment of doxorubicin. The constancy of the ratio suggests that the drug remains in an aqueous environment.
(TIF)

Figure S2 **Measuring the nuclear versus non-nuclear doxorubicin accumulation.** HCT116 monolayers were grown to confluency and then incubated in buffer solution (of desired pH and salt composition). Monolayers were then loaded with Hoechst 33342 and doxorubicin. *(A)* Doxorubicin fluorescence (488 nm excitation) recorded confocally, showing signal in nuclear and non-nuclear regions. *(B)* Hoechst 33342 fluorescence (405 nm excitation) used to identify nuclei. *(C)* Doxorubicin fluorescence in nuclear regions identified on the basis of supra-threshold Hoechst 33342 signal. The ratio of doxorubicin fluorescence in nuclear and non-nuclear regions was determined as (nuclear doxorubicin fluorescence) divided by (total doxorubicin fluorescence minus nuclear doxorubicin fluorescence).
(TIF)

## References

[1] Raghunand N, He X, van Sluis R, Mahoney B, Baggett B (1999). Enhancement of chemotherapy by manipulation of tumour pH.. Br J Cancer.

[2] Gerweck LE, Kozin SV, Stocks SJ (1999). The pH partition theory predicts the accumulation and toxicity of doxorubicin in normal and low-pH-adapted cells.. Br J Cancer.

[3] Gerweck LE, Seetharaman K (1996). Cellular pH gradient in tumor versus normal tissue: potential exploitation for the treatment of cancer.. Cancer Res.

[4] Stubbs M, McSheehy PM, Griffiths JR, Bashford CL (2000). Causes and consequences of tumour acidity and implications for treatment.. Mol Med Today.

[5] Vaupel P, Kallinowski F, Okunieff P (1989). Blood flow, oxygen and nutrient supply, and metabolic microenvironment of human tumors: a review.. Cancer Res.

[6] Raghunand N, Zhang S, Sherry AD, Gillies RJ (2002). In vivo magnetic resonance imaging of tissue pH using a novel pH-sensitive contrast agent, GdDOTA-4AmP.. Acad Radiol.

[7] Smalley RV, Lefante J, Bartolucci A, Carpenter J, Vogel C (1983). A comparison of cyclophosphamide, adriamycin, and 5-fluorouracil (CAF) and cyclophosphamide, methotrexate, 5-fluorouracil, vincristine, and prednisone (CMFVP) in patients with advanced breast cancer.. Breast Cancer Res Treat.

[8] Gerweck LE, Vijayappa S, Kozin S (2006). Tumor pH controls the in vivo efficacy of weak acid and base chemotherapeutics.. Mol Cancer Ther.

[9] Mahoney BP, Raghunand N, Baggett B, Gillies RJ (2003). Tumor acidity, ion trapping and chemotherapeutics. I. Acid pH affects the distribution of chemotherapeutic agents in vitro.. Biochem Pharmacol.

[10] Raghunand N, Mahoney BP, Gillies RJ (2003). Tumor acidity, ion trapping and chemotherapeutics. II. pH-dependent partition coefficients predict importance of ion trapping on pharmacokinetics of weakly basic chemotherapeutic agents.. Biochem Pharmacol.

[11] Noel G, Peterson C, Trouet A, Tulkens P (1978). Uptake and subcellular localization of daunorubicin and adriamycin in cultured fibroblasts.. Eur J Cancer.

[12] Schindler M, Grabski S, Hoff E, Simon SM (1996). Defective pH regulation of acidic compartments in human breast cancer cells (MCF-7) is normalized in adriamycin-resistant cells (MCF-7adr).. Biochemistry.

[13] Lee CM, Tannock IF (2006). Inhibition of endosomal sequestration of basic anticancer drugs: influence on cytotoxicity and tissue penetration.. Br J Cancer.

[14] Boron WF (2004). Regulation of intracellular pH.. Adv Physiol Educ.

[15] Cutts SM, Nudelman A, Rephaeli A, Phillips DR (2005). The power and potential of doxorubicin-DNA adducts.. IUBMB Life.

[16] Cullinane C, Cutts SM, van Rosmalen A, Phillips DR (1994). Formation of adriamycin–DNA adducts in vitro.. Nucleic Acids Res.

[17] Swietach P, Patiar S, Supuran CT, Harris AL, Vaughan-Jones RD (2009). The role of carbonic anhydrase 9 in regulating extracellular and intracellular ph in three-dimensional tumor cell growths.. J Biol Chem.

[18] Swietach P, Wigfield S, Cobden P, Supuran CT, Harris AL (2008). Tumor-associated carbonic anhydrase 9 spatially coordinates intracellular pH in three-dimensional multicellular growths.. J Biol Chem.

[19] Long BH, Wang L, Lorico A, Wang RC, Brattain MG (1991). Mechanisms of resistance to etoposide and teniposide in acquired resistant human colon and lung carcinoma cell lines.. Cancer Res.

[20] Wu L, Smythe AM, Stinson SF, Mullendore LA, Monks A (1992). Multidrug-resistant phenotype of disease-oriented panels of human tumor cell lines used for anticancer drug screening.. Cancer Res.

[21] Aouida M, Poulin R, Ramotar D (2010). The human carnitine transporter SLC22A16 mediates high affinity uptake of the anticancer polyamine analogue bleomycin-A5.. J Biol Chem.

[22] Hulikova A, Vaughan-Jones RD, Swietach P (2011). Dual role of CO2/HCO3(-) formula buffer in the regulation of intracellular pH of three-dimensional tumor growths.. J Biol Chem.

[23] Rzymski T, Milani M, Pike L, Buffa F, Mellor HR (2010). Regulation of autophagy by ATF4 in response to severe hypoxia.. Oncogene.

[24] Thomas JA, Buchsbaum RN, Zimniak A, Racker E (1979). Intracellular pH measurements in Ehrlich ascites tumor cells utilizing spectroscopic probes generated in situ.. Biochemistry.

[25] Gewirtz DA (1999). A critical evaluation of the mechanisms of action proposed for the antitumor effects of the anthracycline antibiotics adriamycin and daunorubicin.. Biochem Pharmacol.

[26] Durand RE, Olive PL (1981). Flow cytometry studies of intracellular adriamycin in single cells in vitro.. Cancer Res.

[27] Luk CK, Tannock IF (1989). Flow cytometric analysis of doxorubicin accumulation in cells from human and rodent cell lines.. J Natl Cancer Inst.

[28] Schneider YJ, Baurain R, Zenebergh A, Trouet A (1979). DNA-binding parameters of daunorubicin and doxorubicin in the conditions used for studying the interaction of anthracycline-DNA complexes with cells in vitro.. Cancer Chemother Pharmacol.

[29] Bryn SR, Dolch GD (1978). Analysis of binding of daunorubicin and doxorubicin to DNA using computerized curve-fitting procedures.. J Pharm Sci.

[30] Okabe M, Unno M, Harigae H, Kaku M, Okitsu Y (2005). Characterization of the organic cation transporter SLC22A16: a doxorubicin importer.. Biochem Biophys Res Commun.

[31] Roninson IB, Chin JE, Choi KG, Gros P, Housman DE (1986). Isolation of human mdr DNA sequences amplified in multidrug-resistant KB carcinoma cells.. Proc Natl Acad Sci U S A.

[32] Karukstis KK, Thompson EH, Whiles JA, Rosenfeld RJ (1998). Deciphering the fluorescence signature of daunomycin and doxorubicin.. Biophys Chem.

[33] Huber V, De Milito A, Harguindey S, Reshkin SJ, Wahl ML (2010). Proton dynamics in cancer.. J Transl Med.

[34] Luciani F, Spada M, De Milito A, Molinari A, Rivoltini L (2004). Effect of proton pump inhibitor pretreatment on resistance of solid tumors to cytotoxic drugs.. J Natl Cancer Inst.

