# Supplementary figures and images for: Importance of Intracellular pH in Determining the Uptake and Efficacy of the Weakly Basic Chemotherapeutic Drug, Doxorubicin
Source: PLoS One. 2012 Apr 26;7(4):e35949. doi: 10.1371/journal.pone.0035949 (PMC3338554; doi:10.1371/journal.pone.0035949)

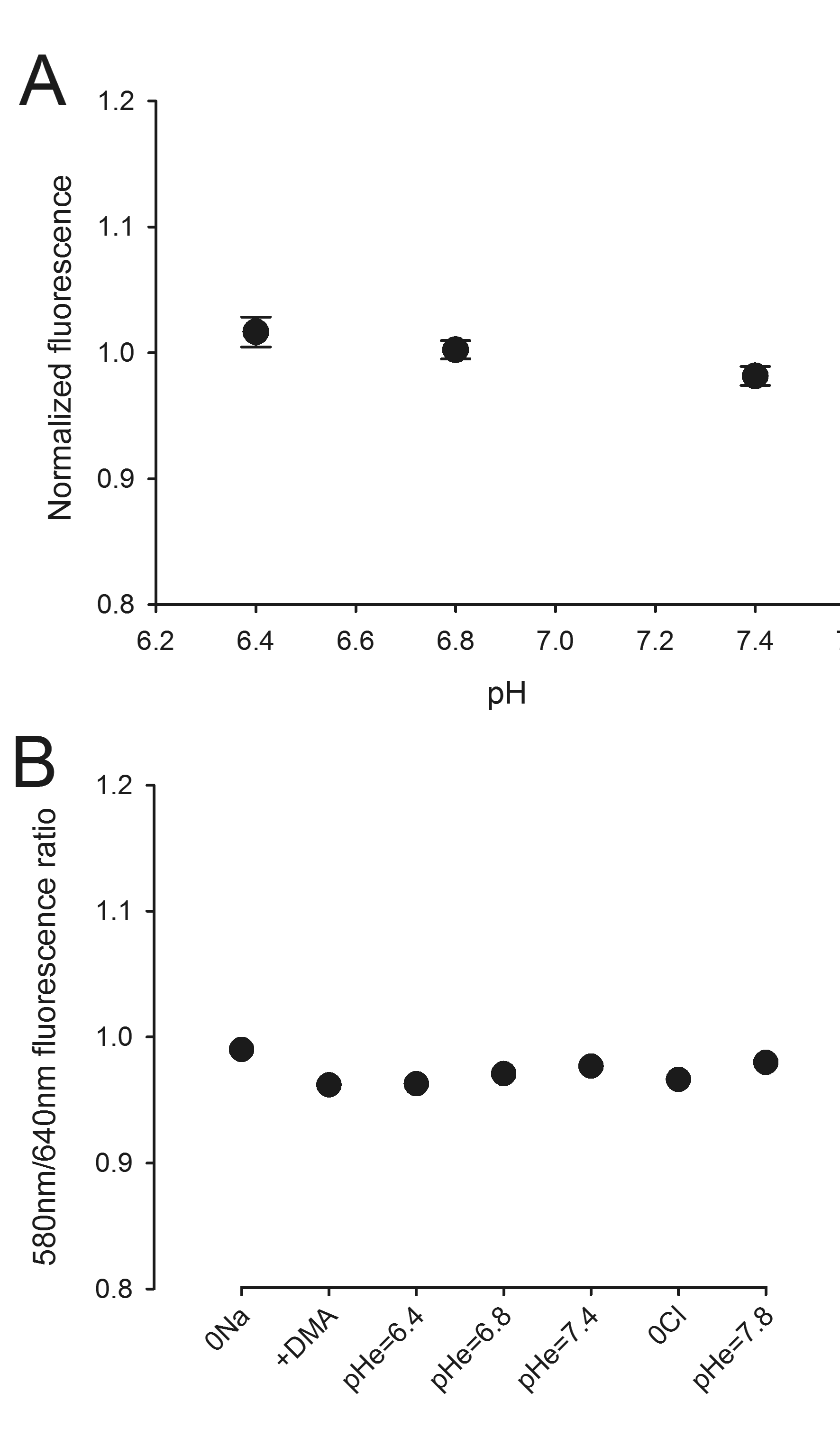

Supplement: Figure S1 — Characterising doxorubicin fluorescence. (A) Doxorubicin (50 µM) fluorescence was measured in Hepes/Mes buffered solution over a range of pH. The pH-sensitivity of fluorescence emission is only mildly pH-sensitivity (<5% per pH unit). (B) Intracellular doxorubicin fluorescence ratio measured flow cytometrically at 580 nm and 640 nm. This ratio carries a signature that describes changes to the ambient environment of doxorubicin. The constancy of the ratio suggests that the drug remains in an aqueous environment. (TIF) [file pone.0035949.s001.tif]

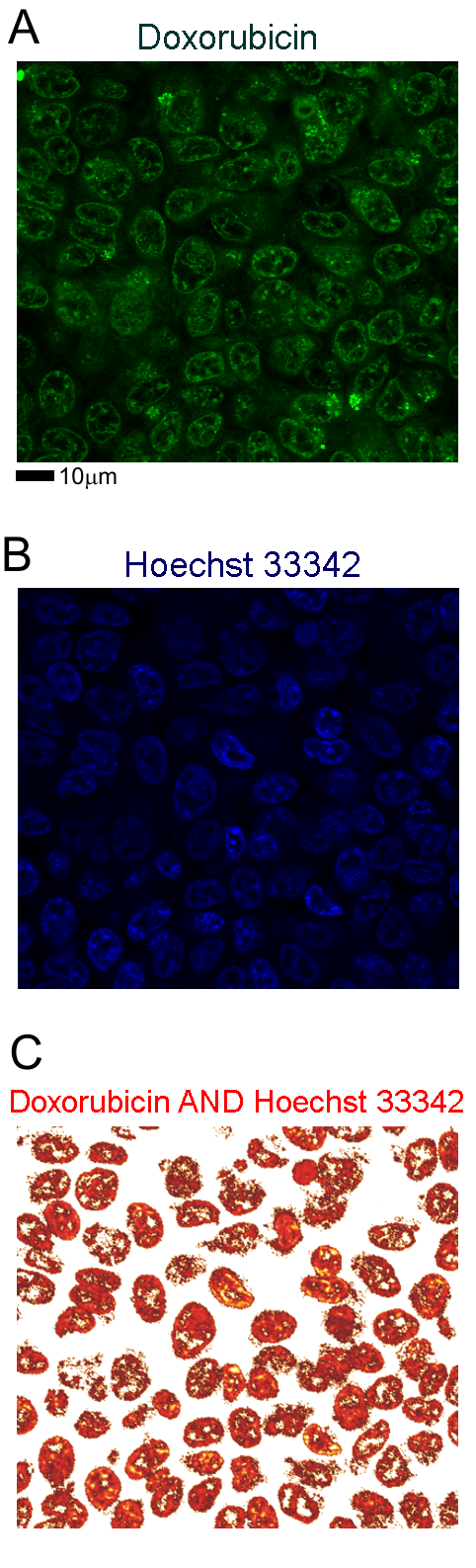

Supplement: Figure S2 — Measuring the nuclear versus non-nuclear doxorubicin accumulation. HCT116 monolayers were grown to confluency and then incubated in buffer solution (of desired pH and salt composition). Monolayers were then loaded with Hoechst 33342 and doxorubicin. (A) Doxorubicin fluorescence (488 nm excitation) recorded confocally, showing signal in nuclear and non-nuclear regions. (B) Hoechst 33342 fluorescence (405 nm excitation) used to identify nuclei. (C) Doxorubicin fluorescence in nuclear regions identified on the basis of supra-threshold Hoechst 33342 signal. The ratio of doxorubicin fluorescence in nuclear and non-nuclear regions was determined as (nuclear doxorubicin fluorescence) divided by (total doxorubicin fluorescence minus nuclear doxorubicin fluorescence). (TIF) [file pone.0035949.s002.tif]
